# Toxicity and Physiological Actions of Carbonic Anhydrase Inhibitors to *Aedes aegypti* and *Drosophila melanogaster*

**DOI:** 10.3390/insects8010002

**Published:** 2016-12-22

**Authors:** Sheena A. M. Francis, Jennina Taylor-Wells, Aaron D. Gross, Jeffrey R. Bloomquist

**Affiliations:** Department of Entomology and Nematology, Emerging Pathogens Institute, University of Florida, Gainesville, FL 32610, USA; sheenafrancis@yahoo.com (S.A.M.F.); jenninataylorwells@gmail.com (J.T.-W.); adgross@ufl.edu (A.D.G.)

**Keywords:** acetazolamide, brinzolamide, dichlorphenamide, dorzolamide, methazolamide insecticide, mode of action

## Abstract

The physiological role of carbonic anhydrases in pH and ion regulation is crucial to insect survival. We examined the toxic and neurophysiological effects of five carbonic anhydrase inhibitors (CAIs) against *Aedes aegypti*. The 24 h larvicidal toxicities followed this rank order of potency: dichlorphenamide > methazolamide > acetazolamide = brinzolamide = dorzolamide. Larvicidal activity increased modestly in longer exposures, and affected larvae showed attenuated responses to probing without overt tremors, hyperexcitation, or convulsions. Acetazolamide and dichlorphenamide were toxic to adults when applied topically, but were of low potency and had an incomplete effect (<50% at 300 ng/mosquito) even after injection. Dichlorphenamide was also the most toxic compound when fed to adult mosquitoes, and they displayed loss of posture and occasionally prolonged fluttering of the wings. Co-exposure with 500 ng of the synergist piperonyl butoxide (PBO) increased the toxicity of dichlorphenamide ca. two-fold in feeding assays, indicating that low toxicity was not related to oxidative metabolism. Dichlorphenamide showed mild depolarizing and nerve discharge actions on insect neuromuscular and central nervous systems, respectively. These effects were increased in low buffer salines, indicating they were apparently related to loss of pH control in these tissues. Overall, sulfonamides displayed weak insecticidal properties on *Aedes aegypti* and are weak lead compounds.

## 1. Introduction

Carbonic anhydrases (CAs) are ubiquitous metalloenzymes that play a fundamental physiological role in regulation of the proton to bicarbonate ratio in cells and tissues by catalyzing the conversion of H_2_O and CO_2_ to H^+^ + HCO_3_^−^ [1]. Their critical role in osmoregulation by rectal glands of mosquito larvae allows for survival in various environmental habitats [2,3,4], as well as the proper maintenance of larval gut pH at 10.5–11.0 [5,6,7]. The presence of these enzymes in the epithelia of female adult mosquitoes [8] and their physiological role after the consumption of a blood meal have also been demonstrated [9].

There are five distinct classes of CAs; α, β, γ, δ, and ζ, which are genetically conserved across the protozoa, as well as plant, and the animal kingdoms [10]. All mammalian CAs are of the α class [11]; however, studies have also reported them in both *Aedes* [6] and *Anopheles* mosquitoes [12]. Additionally, of the five families of CAs, β-CAs are found mainly in invertebrates, which suggests they might be possible targets for selective pesticides [13,14]. A number of CA inhibitors (CAIs) exist, and they are important pharmaceuticals, often used for the oral or topical treatment of glaucoma [15], and show nanomolar affinities, in vitro, for CAs from *Drosophila melanogaster* and *Aedes aegypti* [14]. Accordingly, we examined the CAIs acetazolamide, methazolamide, brinzolamide, dorzolamide, and dichlorphenamide for larvicidal and adulticidal properties against *Aedes aegypti*, as well as their effects on insect nerve and muscle physiology.

## 2. Materials and Methods

### 2.1. Chemicals

The structures of the CAIs used in this study are shown in Table 1. Acetazolamide (>99%), piperonyl butoxide (PBO > 90%), and dorzolamide (>98%) were purchased from Sigma-Aldrich Chemical Company (St., Louis, MO, USA). Methazolamide (>95%) was obtained from Santa Cruz Biotechnology (Santa Cruz, CA, USA.). Brinzolamide (99%) and dichlorphenamide (99%) were acquired from USP Reference Standard (Rockville, MD, USA). Technical permethrin (99%) was purchased from Chem Service, Inc. (West Chester, PA, USA) and propoxur (99%) from Fluka (St. Louis, MO, USA). DMSO (dimethyl sulfoxide) (99.7%) and ethanol (>99%) were obtained from Fisher Bioreagent (Fair Lawn, NJ, USA). Buffer reagents were purchased from various commercial suppliers.

### 2.2. Mosquito Rearing

Early third to fourth instar *Aedes aegypti* larvae were donated by the United States Department of Agriculture, Agricultural Research Service (USDA, ARS, Gainesville, FL, USA). Larvae were harvested in unfiltered tap water and held in trays at 28 °C, under a 12 h light/dark cycle, which was maintained for all stages of development. The larvae were fed three parts liver powder and two parts Brewer’s yeast, both from MP Biomedical (Solon, OH, USA). Pupae were collected and placed in an emergence cage. Adult mosquitoes were maintained at the aforementioned temperature and diurnal cycle, and fed a diet of 10% sucrose solution. For all experiments, third to fourth instar larvae were used or adult females that were one to five days post-emergence.

### 2.3. Mosquito Larval Assays

For intact larvae, all compounds were dissolved in 90% ethanol and 10% dimethyl sulfoxide (DMSO), and serial dilutions were performed into tap water for each test compound (concentration between 250–1000 ppm). Larval assays were performed using 10 third instar-larvae in a Petri dish. Each dish contained 2 mL of water, with controls containing vehicle alone. Mortality data were recorded twenty-four hours post-exposure.

A 5 h headless larval paralysis assay, designed to assess the activity of compounds that do not penetrate well the insect cuticle were also performed essentially as described [17]. Briefly, fourth instar larvae were placed in a Petri dish containing no water, in order to minimize their mobility. The head of larvae was gently pulled away from the body using two pairs of forceps. Immediately after dissection, headless larvae (*n* = 10) were transferred to a Petri dish containing 1 mL of mosquito physiological saline [18] containing 154 mM NaCl, 1.4 mM CaCl_2_, and 2.7 mM KCl, typically with 4 mM HEPES buffer (pH = 6.9). The pH of the saline was tested before each use to ensure correct pH.

### 2.4. Mosquito Adult Assays

Compounds were dissolved and serially diluted in ethanol or a mixture of 90% ethanol + 10% DMSO. Adult female *A*. *aegypti* mosquitoes were briefly anaesthetized on ice, and 0.2 μL of chemical solution was applied to the dorsal thorax using a Hamilton 700 series syringe and a PB600 repeating dispenser (Thermo Fisher Scientific, Hampton, NH, USA). Control treatments with vehicle alone typically gave mortality of less than 10%. After treatment, mosquitoes were kept in paper cups and supplied with 10% sucrose solution for 24 h before mortality was recorded.

Mosquitoes (*n* = 10) were anesthetized on ice, placed in glass tubes, and after recovery, starved in vials for 6 h. The mosquitoes were fed a diet containing 1 mL of CAI dissolved in 10% sucrose solution directly if it was sufficiently water soluble, or a with carrier (3% DMSO or 4% acetone) that was dispersed into 10% sucrose. Mortality data were recorded twenty-four hours post exposure. For synergism tests in feeding assays, 500 ng of PBO dissolved in ethanol was applied topically to the mosquitoes 4 h prior to conducting the feeding assay.

For measurements of toxicity via injection, each compound was dissolved in a 90% ethanol + 10% DMSO mixture and serially diluted into mosquito saline [18]. Mosquitoes were anesthetized on ice and placed on their sides. A 0.2 µL aliquot of each compound was administered with a glass capillary needle attached to a manual micro-syringe pump with accompanying gas-tight Luer tip syringe (World Precision Instruments, Inc., Sarasota, FL, USA). The test compound was injected into the thorax of each mosquito. After treatment, mosquitoes (*n* = 10) were kept in paper cups and supplied with 10% sucrose solution for 24 h before mortality was recorded.

### 2.5. Toxicity Data Analyses

Topical, feeding, and injection treatments were performed in triplicate and reported as the mean of replicated toxicity determinations, and by probit log 10 analysis in the statistical analysis package SAS 9.3 where appropriate (SAS Institute, Cary, NC, USA). Statistical analysis was performed and graphics were generated using GraphPad Prism v7.0 (GraphPad Software, La Jolla, CA, USA). To compare the toxicity of the chemicals used in each bioassay, a one-way analysis of variance (ANOVA) was used, along with a Dunnett’s multiple-comparison or *t*-test to compare control group with a treated group, where significance (α = 0.05) was observed. 

### 2.6. Neuromuscular Electrophysiology

Neuromuscular recordings with dichlorphenamide were made from fourth instar *Aedes aegypti* larvae. Larvae were pinned to a silicone dish in a bath containing 500 µL Hayes’ [18] insect saline containing 0.42 mM (1/10th buffer strength), 4.2 mM HEPES or 1.2 mM NaHCO_3_ with a pH 6.9. Larvae were incised longitudinally and pinned open, the gut and central nerve cord was then removed to reveal the abdominal muscle sheet. Membrane potential was recorded via a glass capillary filled with 1 M KCl, which was placed in a large fiber of ventrolateral muscle II [19]. The signal was amplified via an Axoclamp 900A (Molecular Devices, Sunnyvale, CA, USA), before filtering through a Hum Bug noise eliminator (A-M Systems, Sequim, WA, USA) and digitized using LabChart 7 (ADInstruments PowerLab 4/30, Colorado Springs, CO, USA), which also included a 50 Hz low pass digital filter. The preparation was left for 5 min to stabilize the membrane potential before the addition of 0.5 µL DMSO (0.1% DMSO (*v*/*v*) final) as a control, followed by various concentrations of dichlorphenamide in DMSO. Treatments were added at 5 min intervals until the final concentration of 100 µM, when the recording was continued for a further 30 min. A minimum of three larvae were used for each experimental condition, with four test concentrations per experiment.

### 2.7. Extracellular Electrophysiology of Drosophila Melanogaster CNS

Extracellular electrophysiology recordings were performed on the central nervous system (CNS) of wandering third-instar *Drosophila melanogaster* larvae [20], collected form a colony of the wild type Oregon R strain, maintained in the Emerging Pathogens Institute at the University of Florida. The CNS was excised from the larvae and placed into a dish containing 1 mL of physiological saline (157 mM NaCl, 3 mM KCl, 2 mM CaCl_2_, 4 mM HEPES). Experiments were initially performed with 4 mM HEPES (normal concentration in saline); then the concentration of HEPES was decreased 90% (0.4 mM). The CNS was transected posterior to the cerebral lobes to disrupt the blood-brain barrier and enhance the penetration of CAIs. Electrical activity was monitored using a suction electrode of peripheral nerves, with amplification of signals by an AC/DC differential amplifier (Model 3000l A-M Systems, Inc., Carlsborg, WA, USA). Descending electrical activity was subjected to window amplitude discrimination and converted into a rate plot, expressed in Hertz (Hz), using LabChart 7 Pro software (ADInstruments Inc., Colorado Springs, CO, USA). Noise (60 Hz) was eliminated using a Hum Bug (A-M Systems, Sequim, WA, USA). Activity was monitored for several minutes to establish a baseline CNS firing rate, and then the test solution (1 µL) was added to the bath and mixed using a 200 µL pipette several times. The final concentration of vehicle (DMSO) was 0.1%. Recording of each concentration was performed for 30 min; a new CNS preparation was used for each concentration and replicate. The CNS firing frequency was averaged over 3-min intervals, immediately prior to the application of the tested compound (baseline) and every three minutes after the application of test compound for 30 min. The average firing rate was plotted in GraphPad Prism v7 (La Jolla, CA, USA). An unpaired *t*-test between individual treatments was performed to determine significant difference at discrete time points following treatment (α = 0.05).

## 3. Results

### 3.1. Larval Bioassay Results

Under the conditions of this assay, acetazolamide, brinzolamide, and dorzolamide were of low toxicity to intact *Aedes* *aegypti* larvae, and caused <50% larval mortality at 1000 ppm in both 24 and 48 h exposures. However, better activity was observed for methazolamide and dichlorphenamide. The methazolamide 24 h LC_50_ was 724 (628–841) and that of dichlorphenamide was 397 (357–474) ppm, where numbers in parentheses indicate 95% confidence limits. Increasing the exposure time to 48 h slightly increased toxicity, resulting in LC_50_ values of 639 (556–727) ppm and 241 (194–291) ppm for methazolamide and dichlorphenamide, respectively. For comparison, propoxur was used as a positive control, and had a 24 h LC_50_ = 0.72 (0.62–0.85) ppm, similar to the 0.6 (0.5–0.7) ppm value reported by Larson et al. [21].

Aspartate was used a positive chemical control in a 5 h headless larvae assay, and the concentration that caused 50% paralysis (PC_50_) was 12 (10–16) ppm, similar to the 4.2 ppm (1.3–13) PC_50_ reported previously [17]. In this assay, only dichlorphenamide was tested because it was the most active CAI against intact larvae and served as a representative sulfonamide in this and all subsequent studies. Control larvae responded in this assay with repeated, vigorous swimming motions when probed with a needle. Exposure of headless larvae to 1000 ppm dichlorphenamide resulted in a relaxed, extended larval posture, and only a single twitch response was elicited by probing. The PC_50_ for dichlorphenamide was 283 (220–480) ppm in 4 mM HEPES buffer, indicating that removal of the cuticular barrier did not greatly improve activity.

### 3.2. Adult Bioassay Results

The CAIs had low toxicity via the topical route, routinely showing <50% mortality at doses up to 1000 ng for acetazolamide and 6000 ng for dichlorphenamide, the latter of which was more soluble in topical application vehicle and could be tested at greater doses. The adulticide permethrin was used as a positive control in these studies and had an LD_50_ of 0.083 (0.063–0.110) ng/mg for topical application. This result was similar to the value of 0.028 ng/mg published previously [22], and indicated that the low toxicity of CAIs was not a consequence of methodology.

Dichlorphenamide was the only CAI that displayed significant toxicity in feeding assays; whereas, the other four compounds caused little or no toxicity at 1000 ppm. With dichlorphenamide, the LC_50_ at 24 h was 1099 (1033–1151) ppm. Intoxicated mosquitoes were observed to enter a reversible knockdown state, depending upon dose. The insects laid on their backs, and displayed bouts of wing fluttering often coupled to a rapid spinning motion. To see if toxicity could be improved by blocking oxidative metabolism, a dose of 500 ng PBO was topically applied to mosquitoes 4 h prior to feeding with dichlorphenamide, and an increase in toxicity was observed, with an LC_50_ = 486 (310–684) ppm, and a synergistic ratio of 2.2. In this same assay, permethrin had an LC_50_ of 15 (12–18) ppm, not too dissimilar from the reported value of 5 ppm for the New Orleans reference strain of *Aedes aegypti* [23].

**Figure 1:**
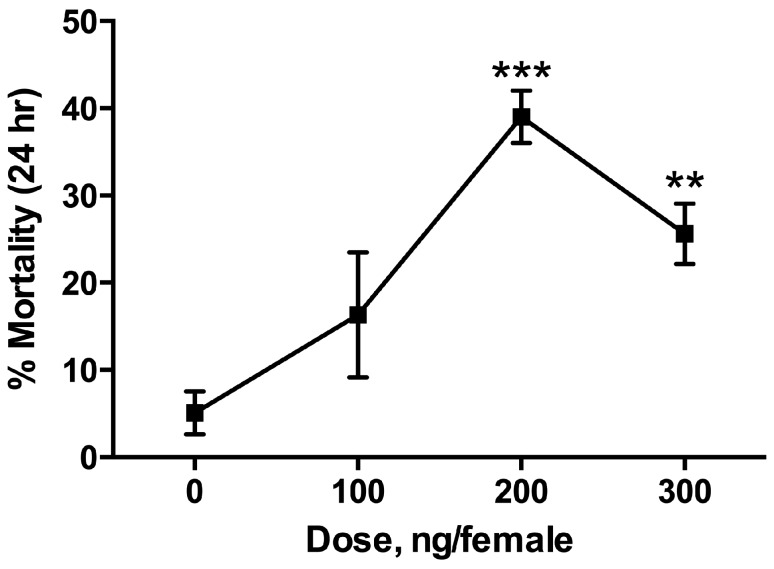
Dose-response relationship for intrathoracic injection of acetazolamide into adult female *Aedes aegypti*. Statistical significance was determined by *t*-test comparison to control mortality, where ** *p* < 0.01 and *** *p* < 0.001.

When injected into the thoracic cavity of mosquitoes, CAIs showed weak toxicity, with maximum mortality of 20%–40% at concentrations of 100 ng/mg–200 ng/mg for dichlorphenamide and acetazolamide. Although the effects of injected CAIs were statistically significant and dose-dependent, mortality did not increase above approximately 40%, even at a dose of 300 ng (Figure 1). To verify the adequacy of the experimental techniques employed for injection toxicity testing, propoxur treated with these methods had an LD_50_ = 0.20 (0.15–0.25) ng/mg. This value is nearly identical to what was found previously; 0.24 (0.16–0.30) ng/mg for *Anopheles gambiae* adults, by Mutunga et al. [24].

### 3.3. Neuromuscular Recordings

**Figure 2:**
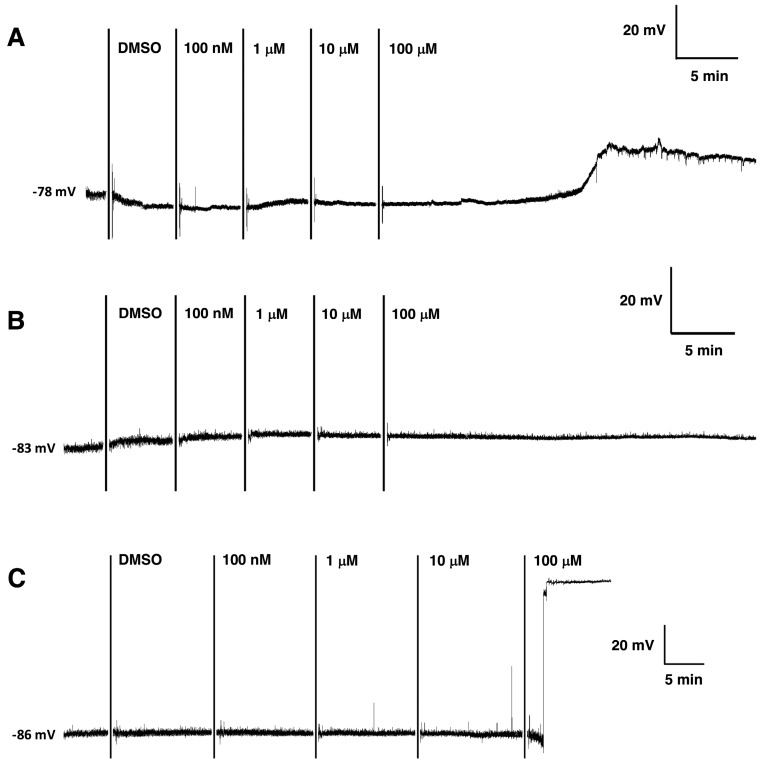
Effects of dichlorphenamide on the membrane potential of *A. aegypti* fourth instar larval muscle. (**A**) In the presence of 1.2 mM bicarbonate; (**B**) in the presence of 4.2 mM HEPES; and (**C**) in the presence of 0.42 mM HEPES (1/10th buffer strength). Each recording shows the membrane potential at the start of the experiment, and treatment additions are indicated by vertical lines. DMSO, dimethyl sulfoxide.

Electrophysiological experiments were conducted on insect neuromuscular and central nervous systems using dichlorphenamide and various buffer compositions to investigate the mode of action. The addition of 100 µM dichlorphenamide in the presence of insect saline containing bicarbonate resulted in a weak depolarizing effect on the membrane potential of *A. aegypti* larval muscle (Figure 2A). This result occurred in four out of five preparations.

There was little or no effect on membrane potential (four out of four preparations) from 100 µM dichlorphenamide treatment in larval saline containing 4.2 mM HEPES (Figure 2B). However, in the presence of insect saline containing 1/10th strength HEPES buffer (0.42 mM), some visible twitching of the larval muscle and spontaneous firing was observed following the addition of 1–10 µM dichlorphenamide. The addition of 100 µM dichlorphenamide then initiated a fast depolarization of the membrane directly to 0 mV (Figure 2C), which was observed in three out of four preparations. The use of 0.1% DMSO and insect saline with reduced HEPES buffer (0.42 mM) did not induce these effects or change the stability of control recordings.

### 3.4. CNS Recordings

**Figure 3:**
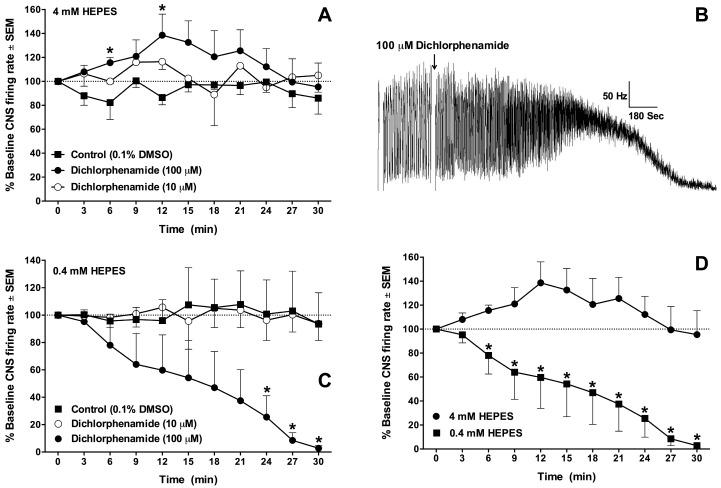
Central nervous system (CNS) firing rate sampled every three minutes after treatment with dichlorphenamide. (**A**) Effects at two concentrations of dichlorphenamide in normal buffer containing 4 mM HEPES; (**B**) Spike rate recording of descending activity in a *D. melanogaster* larval CNS after treatment with 100 μM dichlorphenamide in 0.4 mM HEPES buffered saline; (**C**) Time course plot of 0.4 mM HEPES data. In (**A**,**C**), an asterisk indicates a statistically significant difference between the dichlorphenamide treatment and control using an unpaired *t*-test (α = 0.05). Numbers in parentheses indicate the concentration of dichlorphenamide in micromolar; (**D**) Replot of 100 μM dichlorphenamide curves in 0.4 and 4 mM HEPES buffers, where an asterisk indicates a statistically significant difference between the dichlorphenamide effects at a given time interval, as described above.

The effect of the carbonic anhydrase inhibitor dichlorphenamide on the firing rate of the larval CNS from *D. melanogaster* was investigated at 10 µM and 100 µM. The CNS firing rate was monitored for 30 min after the addition of drug or vehicle control at two buffer concentrations (4 mM and 0.4 mM). Dichlorphenamide at 10 µM did not have a statistically significant effect during a 30 min application compared to the control at 4 mM HEPES (Figure 3A). Dichlorphenamide at 100 µM (4 mM HEPES) increased nerve firing within 12 min after treatment, followed by a return to the baseline firing rate (Figure 3A). The increase in nerve firing was significant at two time points (6 min and 12 min) compared to control (Figure 3A). The effect of dichlorphenamide on the firing rate of the CNS was also assessed at one-tenth concentration of HEPES (0.4 mM; Figure 3B,C).

A recording of the spike rate in the presence of 100 µM dichlorphenamide is shown in Figure 3B, where the rhythmic discharge is transformed into constant firing that subsides to near zero over the ensuing 30 min observation period. The CNS firing rate was significantly decreased 24 min after treatment with 100 µM dichlorphenamide (Figure 2C); whereas 10 µM dichlorphenamide did not show a statistically significant decrease in the CNS firing rate compared to the control (Figure 2C) at any time point during the recording. There were statistically significant effects of 100 µM dichlorphenamide at nearly all time points when comparing the two buffer concentrations (*p* < 0.05), as shown in Figure 3D.

## 4. Discussion

The compounds tested had varying toxic effects on *A. aegypti* larvae, and were much less efficacious than the commercial insecticides propoxur and permethrin. Moreover, positive controls run with these insecticides validated the methods used to test for CAI toxicity in the various experimental paradigms. Previous reports demonstrated that sulphonamide CAIs possessed toxicity to mosquito larvae. Rocha et al. [25] tested acetazolamide, dorzolamide, ethoxzolamide, and methazolamide against *Anopheles albimanus* larvae. They found that in tests running up to 72 h, none of the compounds exceeded 50% mortality at 50 ppm, except ethoxzolamide, which caused >80% mortality within 24 h. Similarly, del Pilar Corena et al. [7] tested methazolamide and acetazolamide on larvae of six different mosquito species. Typical LC_50_ values in this study are reflected in data for *Aedes aegypti*, where methazolamide and acetazolamide were 75 and 70 ppm, respectively. In the present study, CAIs showed less potent lethal activity against third instar *A. aegypti* larvae by several fold, but our experiments differed from the methods of del Pilar Corena et al. [7], who used mixed groups of first to fourth instar larvae. We did observe that methazolamide showed greater larvicidal toxicity than acetazolamide, corroborating the results of del Pilar Corena et al. [7].

It was disappointing that CAIs, which are often administered topically as drugs [15], had little effect on adult mosquitoes when applied topically, or even when injected. These observations were surprising, considering that Fisher et al. [26] showed that methazolamide bound tightly to the catalytic site of *A. aegypti* carbonic anhydrase I (*Aa*CAI), with a K_i_ of 2.7 ± 0.3 nM. CAs are expressed in the midgut of adult mosquitoes, where they play an important role in regulating pH [8] and feeding studies with adults gave the most consistent toxic responses. Dichlorphenamide was more effective than the other CAIs used here, and was the most toxic compound on *A. aegypti* both as a larvicide and in adult feeding assays. Pre-exposure treatment with 500 ng PBO increased the toxicity of dichlorphenamide ca. two-fold in feeding assays, indicating that the low toxicity was not obviously related to oxidative metabolism. The relatively polar nature of the CAIs and their p*K*a values (Table 1, [16]) would explain the low contact activity against adults, and low bioavailability was probably a rate limiting factor in toxicity. However, there was not a large increase in toxicity in headless larvae assays, and injection into adults increased toxicity, but did not result in uniform lethality, even at a dose of 300 ng/mg, at or near the limit of vehicle that the mosquitoes could tolerate. These findings indicate that other unknown processes are affecting toxicity.

The observed knockdown behavior with dichlorphenamide and the paralytic effects in the headless larvae assay suggested that in addition to midgut toxicity, dichlorphenamide may affect nervous or muscular activity of the insect. Intracellular measurements of muscle membrane potential showed depolarization that was enhanced by reduced levels of buffer, supporting the conclusion that blockage of CA and subsequent imbalance in cellular pH was a likely mechanism. The twitching observed at lower concentrations of dichlorphenamide (Figure 2C) indicated that other effects (possibility presynaptic) might be occurring. Similarly, there was an increase in nerve firing discharge rate in larval *D. melanogaster* CNS, followed by a decline. A depolarizing effect of dichlorphenamide on the nerve membrane could be responsible for both effects; release of transmitters occurring early in poisoning that segues into block as the nerve is progressively depolarized. Both neuromuscular and central effects of dichlorphenamide required a concentration of 100 μM, consistent with the relatively large doses required to observe toxicity, in vivo.

## 5. Conclusions

As shown previously, a subset of CAIs were lethal to larval *Aedes aegypti*, showing toxicity in intact larvae over 24 h, and also paralysis within 5 h in headless larvae. Adult females showed little effect after high topical doses, but toxicity was observed in feeding assays and by injection. The overall acute toxic potency of the CAIs was orders of magnitude less than established insecticides, which is typical for new lead compounds. The CAI dichlorphenamide caused a depolarization of mosquito larval muscle and biphasic elevation and depression of *D. melanogaster* larval CNS firing; all effects were enhanced by lowering the buffer concentration of the insect saline. The buffer dependence of these effects supports the conclusion that the physiological actions in nerve and muscle are related to CA inhibition.

## Figures and Tables

**Table 1:** Chemical structures and physical characteristics of the carbonic anhydrase (CA) inhibitors (CAIs) tested in this study. The p*K*_a_ values were experimentally determined (calculated for brinzolamide) and logP values calculated based on neural network algorithms [16].

CAIs	Structure	p*K*_a_	IA Log P
Acetazolamide	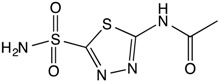	7.4	−0.25
Brinzolamide	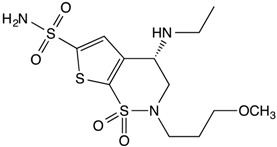	7.2	0.22
Dichlorphenamide	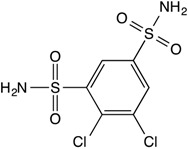	8.3	−0.04
Dorzolamide	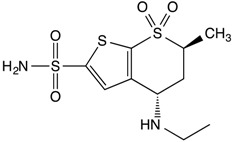	8.4	0.71
Methazolamide	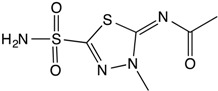	7.2	−0.08
